# Cerebral Microbleeds in Different Brain Regions and Their Associations With the Digital Clock-Drawing Test: Secondary Analysis of the Framingham Heart Study

**DOI:** 10.2196/45780

**Published:** 2024-07-29

**Authors:** Samia C Akhter-Khan, Qiushan Tao, Ting Fang Alvin Ang, Cody Karjadi, Indira Swetha Itchapurapu, David J Libon, Michael Alosco, Jesse Mez, Wei Qiao Qiu, Rhoda Au

**Affiliations:** 1 Department of Global Health & Social Medicine King's College London London United Kingdom; 2 Framingham Heart Study Boston University School of Medicine Boston, MA United States; 3 Pharmacology, Physiology & Biophysics Boston University School of Medicine Boston, MA United States; 4 Department of Anatomy & Neurobiology Boston University School of Medicine Boston, MA United States; 5 Department of Epidemiology Boston University School of Public Health Boston, MA United States; 6 Slone Epidemiology Center Boston University School of Medicine Boston, MA United States; 7 Department of Geriatrics and Gerontology Rowan University Glassboro, NJ United States; 8 Department of Psychology New Jersey Institute for Successful Aging, School of Osteopathic Medicine Rowan University Glassboro, NJ United States; 9 Department of Neurology Boston University School of Medicine Boston, MA United States; 10 Alzheimer’s Disease and Chronic Traumatic Encephalopathy Centers Boston University Boston, MA United States; 11 Department of Psychiatry Boston University School of Medicine Boston, MA United States

**Keywords:** cerebral microbleeds, CMB, digital clock-drawing test, DCT, Alzheimer disease, dementia, early screening, Boston Process Approach, cerebral microbleed, neuroimaging, cerebrovascular diseases, aging, MRI, magnetic resonance imaging, clock-drawing test, cognitive function

## Abstract

**Background:**

Cerebral microbleeds (CMB) increase the risk for Alzheimer disease. Current neuroimaging methods that are used to detect CMB are costly and not always accessible.

**Objective:**

This study aimed to explore whether the digital clock-drawing test (DCT) may provide a behavioral indicator of CMB.

**Methods:**

In this study, we analyzed data from participants in the Framingham Heart Study offspring cohort who underwent both brain magnetic resonance imaging scans (Siemens 1.5T, Siemens Healthcare Private Limited; T2*-GRE weighted sequences) for CMB diagnosis and the DCT as a predictor. Additionally, paper-based clock-drawing tests were also collected during the DCT. Individuals with a history of dementia or stroke were excluded. Robust multivariable linear regression models were used to examine the association between DCT facet scores with CMB prevalence, adjusting for relevant covariates. Receiver operating characteristic (ROC) curve analyses were used to evaluate DCT facet scores as predictors of CMB prevalence. Sensitivity analyses were conducted by further including participants with stroke and dementia.

**Results:**

The study sample consisted of 1020 (n=585, 57.35% female) individuals aged 45 years and older (mean 72, SD 7.9 years). Among them, 64 (6.27%) participants exhibited CMB, comprising 46 with lobar-only, 11 with deep-only, and 7 with mixed (lobar+deep) CMB. Individuals with CMB tended to be older and had a higher prevalence of mild cognitive impairment and higher white matter hyperintensities compared to those without CMB (*P*<.05). While CMB were not associated with the paper-based clock-drawing test, participants with CMB had a lower overall DCT score (CMB: mean 68, SD 23 vs non-CMB: mean 76, SD 20; *P*=.009) in the univariate comparison. In the robust multiple regression model adjusted for covariates, deep CMB were significantly associated with lower scores on the drawing efficiency (β=–0.65, 95% CI –1.15 to –0.15; *P*=.01) and simple motor (β=–0.86, 95% CI –1.43 to –0.30; *P*=.003) domains of the command DCT. In the ROC curve analysis, DCT facets discriminated between no CMB and the CMB subtypes. The area under the ROC curve was 0.76 (95% CI 0.69-0.83) for lobar CMB, 0.88 (95% CI 0.78-0.98) for deep CMB, and 0.98 (95% CI 0.96-1.00) for mixed CMB, where the area under the ROC curve value nearing 1 indicated an accurate model.

**Conclusions:**

The study indicates a significant association between CMB, especially deep and mixed types, and reduced performance in drawing efficiency and motor skills as assessed by the DCT. This highlights the potential of the DCT for early detection of CMB and their subtypes, providing a reliable alternative for cognitive assessment and making it a valuable tool for primary care screening before neuroimaging referral.

## Introduction

It is widely shown that cerebrovascular diseases increase the risk of Alzheimer disease (AD) and other dementia [1,2]. Cerebral microbleeds (CMB) are one of such cerebrovascular abnormalities, defined as small chronic brain hemorrhages, likely caused by structural abnormalities of the small vessels of the brain [3]. The prevalence of CMB is estimated to be as high as 34% in people with ischemic stroke and 60% in people with nontraumatic intracerebral hemorrhage [4]. CMB have also been associated with cognitive impairment and increased risk for AD development across multiple studies [5,6]. CMB can be divided into 2 subclasses based on their location in the brain, that is, lobar and deep CMB. A recent meta-analysis reported a 75% increased risk of dementia with deep or mixed CMB [7].

In light of population aging demographics, these figures are concerning, and solutions for early detection of emergent disease and AD risk factors at a preclinical stage to prevent the disease’s development are urgently needed [8]. Neuroimaging methods, such as brain magnetic resonance imaging (MRI) and computerized axial tomography scans, are valuable tools to detect cerebrovascular pathology. Currently, lobar and deep CMB can only be identified by brain MRI. However, these imaging tools are costly and, in most cases, not accessible in rural areas and low-income contexts. There are over 50 million people estimated to live with dementia and AD worldwide, with the highest increases in lower- and middle-income countries [9]. The worldwide costs of dementia are estimated to amount to over US $1 trillion [10]. To drive down the costs and promote early detection of early brain pathology for AD risk, including CMB, which have subtle clinical symptoms, one promising approach is to explore new, inexpensive technologies such as digital neuropsychological assessments coupled with machine learning analytics to detect and screen for cerebrovascular diseases in clinic before applying neuroimaging tools and neuropsychological assessments [11].

The clock-drawing test (CDT; traditional, paper based) is an easily applicable cognitive test, and prior research has documented that patients with dementia with MRI evidence of vascular disease make more clock-drawing errors than other groups [12-14]. However, the traditional analog, manually scored CDT is limited by its low sensitivity and specificity for different brain diseases, especially at an early stage or where there is subtle brain pathology. Moreover, analog scoring systems are only able to generate a limited number of clock-drawing metrics [15]. Developing efficient tools to measure cognitive change and brain health, especially in the preclinical stage of AD, is necessary. Recently, the CDT has been transformed through the use of a digital ballpoint pen, replacing a conventional ink pen, and coupled with a dot pattern that provides raw, time-stamped data that capture the full performance sequence (digital clock-drawing test; DCT), thus, generating large, detailed data on cognition that cannot be derived using the traditional CDT [11]. Recently, the Framingham Heart Study (FHS) analyzed DCTs from 1833 participants using machine learning to detect cognitive nuances. The DCT showed superiority over existing methods such as the Mini Mental Status Examination in detecting early cognitive impairment and characterizing individuals along the AD trajectory [16]. In addition, the DCT has evidenced its diagnostic value for early screening for mild cognitive impairment (MCI) and AD [17-19]. Further, the DCT was also associated with biomarkers relevant to cognitive impairment and AD, collected by positron emission tomography imaging, for example, amyloid and τ pathology [20].

Given the important role of CMB in the development of AD, the value of operationalizing putative negative effects on behavior caused by CMB using less costly and more accessible diagnostic tools is evident. Using data from the FHS, we predicted that the DCT would be useful in detecting CMB in older adults without dementia. We also examined whether the DCT could detect CMB in different brain regions, for example, lobar and deep CMB.

## Methods

### Study Sample

FHS is a multigeneration, community-based, prospective cohort study in Framingham, Massachusetts. The FHS offspring cohort (Generation 2) has been longitudinally examined in 9 core examinations, with examinations occurring on average every 4 years between 1971 and 2014. Details about this cohort have been previously described elsewhere [21]. These participants also had serial neuropsychological and MRI scans on average every 5 years between 1999 and 2019 [22]. For this study, FHS Generation 2 participants who were over 45 years old and had brain MRI CMB data (2000-2009) and DCT assessments (2011-2018; n=1072) were included. For primary analyses, participants were excluded if they had dementia (n=23) or had a history of stroke (n=29).

### Clock-Drawing Measures: the CDT Versus DCT

The manual for administering and scoring the CDT has been previously described [23]. The CDT score using this analog (hand-scored) method ranged from 0 (no abnormality) to 3 (severe impairment). The DCT was obtained using the digital pen technology from Anoto, and the time-stamped features were processed by Linus Health Inc. Similar to the original paper-pencil CDT, the DCT contains a command and a copy condition [20]. In brief, the DCT contains multiple objective measurements that were derived from approximately 5000 digital clock drawings using machine learning algorithms to precisely evaluate nuances in performance beyond successful task completion [24]. Variables from the command and copy versions were combined from the machine learning calculations into an overall command or copy score ranging from 0 to 100. A similar technique was used for the domain-specific subscores measuring drawing efficiency, information processing, simple motor, and spatial reasoning (see previous publications [20,25] and Table S1 in Multimedia Appendix 1 for details).

### Brain MRI

The FHS MRI protocol and CMB diagnosis criteria have been previously described [26]. Briefly, participants were imaged by a Siemens 1.5T MRI, using a 3D T1-weighted coronal spoiled gradient-recalled echo sequence. All images were transferred to and processed by the University of California Davis Medical Center without knowledge of clinical information. The MRI scans were conducted between 2000 and 2009 with gradient recalled echo T2-weighted sequences, allowing for the detection of CMB. Using recently published guidelines [27], CMB were defined as rounded or ovoid hypointense lesions on a T2*-GRE weighted sequence, measuring <10 mm in diameter and surrounded by brain parenchyma over at least half the circumference of the lesion. The presence, number, and location of CMB were determined. Reliability measures for CMB readings have previously been described [28,29]. In line with previous studies [29], CMB location in the brain was classified into subgroups based on assumed pathophysiology (cerebral amyloid angiopathy and hypertensive vasculopathy), and it was classified into 2 locations: deep and lobar, with each participant potentially having 1 or more CMB. All participants were grouped into 4 subgroups based on assumed pathophysiology (cerebral amyloid angiopathy and hypertensive vasculopathy), which included the no CMB (control) group and groups with lobar-only, deep-only, and mixed (deep+lobar) CMB. White matter hyperintensities (WMHs) were segmented with fluid-attenuated inversion recovery and gray matter with T1-weighted images by semiautomated procedures, as previously described [30]. WMHs were adjusted for head size (by dividing WMHs by the total cerebral volume).

### Statistical Analysis

Baseline characteristics of study participants were evaluated for the total sample and for CMB status. To compare the CMB status groups, 2-tailed *t* tests were applied for continuous variables, and *χ*^2^ tests or Fisher exact tests were applied for categorical variables. To facilitate standardized comparisons between different scales in the models adjusted for covariates, the digital clock variables were transformed to *z* scores (mean 0, SD 1), for example, the overall DCT score (percentage range 0%-100%) was rescaled to a *z* score after logit transformation; all other DCT scores were also rescaled to *z* scores to standardize the variables that have different value ranges. Robust multivariable linear regression models were applied to assess whether the DCT scores (outcomes variables) were significantly different between different CMB subgroups and no CMB (control) group. All models were adjusted for age, sex, education, WMHs, and the time difference between brain MRI and DCT. A sensitivity analysis was conducted by including subjects with dementia (n=23), or a history of stroke (n=29) that were excluded from the main models. To test whether the DCT facet scores could distinguish different CMB subgroups from no CMB (control group), we also calculated the area under the curve (AUC) of a receiver operating characteristic (ROC) curve analysis based on multinomial classification models that were adjusted for main confounding factors such as age, sex, time between examinations, and MCI. The AUC, ranging from 0.5 to 1, is a key metric for evaluating a classifier’s ability to distinguish between positive and negative outcomes. A value nearing 1 indicates a highly accurate model, while an AUC of 0.5 suggests performance equivalent to random chance, indicating no predictive power. The results were shown as beta estimates (β) with 95% CIs. Statistical significance was indicated by a *P* value <.05 (2-tailed tests). All statistical analyses were performed using R (version 4.2.1; R Foundation for Statistical Computing).

In our data analysis, we used several R packages and functions. Specifically, we used the *rlm* function from the *MASS* library for robust linear model fitting [31], the *lmrob* function from the *robustbase* library for MM-type estimator calculation in linear regression [32], and the *sandwich* library for robust SE estimation in nonlinear models [33]. Additionally, the *pROC* library facilitated ROC curve analysis and AUC value calculation [34].

### Ethical Considerations

The study was conducted per the Declaration of Helsinki, and ethical approval was provided by Boston University’s Institutional Review Board (H-40620). All participants provided informed consent, and data was de-identified.

## Results

The 1020 participants from the FHS Generation 2 sample were on average aged 72 (SD 7.9) years, and 57.35% (n=585) of them were female (Table 1). Among them, 64 (6.27%) participants had at least 1 CMB. Participants with CMB were more likely to be older (*P*<.001), have MCI (*P*=.02), and have greater WMHs (*P*<.001). There were no differences between the participants with and without CMB for the traditional, analog-scored CDT, in either the command or copy condition. By contrast, participants with CMB showed a significantly lower overall combined command or copy DCT score (*P*=.01) than those without CMB (Table 1).

Table 1 shows that participants with CMB showed worse performance on several command and copy DCT domains. Specifically, participants with CMB scored lower on the command spatial reasoning (*P*=.03), copy drawing efficiency (*P*=.03), and information processing (*P*=.01) subscales. We further divided participants with CMB into those who had CMB exclusively in lobar regions (46/64, 72%), those who had CMB only in deep regions (11/64, 17%), and those who had mixed lobar+deep CMB (7/64, 11%) and used the DCT domain score to examine CMB (Table 2). After adjusting for covariates, there were no statistically significant differences in the overall DCT or domain scores between participants with any CMB (*P*>.05), compared to those without CMB. However, when analyzing the subscales, participants with deep-only CMB had lower scores on the command simple motor subscale than the reference group (β=–0.86, 95% CI –1.43 to –0.30; *P*=.003). Additionally, participants with mixed (lobar+deep) CMB had lower scores on the command spatial reasoning subscale than the reference group (β=–1.70, 95% CI –2.29 to –1.11; *P*<.001). In contrast, participants with lobar CMB did not show associations with any domain score under both command and copy conditions of the DCT. The results were similar when including participants with dementia or stroke in the analysis (Table S2 in Multimedia Appendix 1).

Robust multivariate linear regression models were applied with DCT scores (overall score and domains) and CMB subtypes (lobar-only, deep-only, and any deep). The reference group were participants without CMB (n=956). The overall DCT score (percentage) was rescaled to a *z* score (mean 0, SD 1) after logit transformation and other DCT scores were rescaled to *z* scores (mean 0, SD 1) after logit transformation. Participants with dementia or stroke were excluded from the analysis (see sensitivity analysis with inclusion in Table S2 in Multimedia Appendix 1). All models were adjusted for age, sex, education, MCI, WMHs, and the time difference between brain MRI and the DCT. The results are shown as standardized β coefficients (β) with 95% CI. *P* values for statistical significance are indicated.

**Figure 1:**
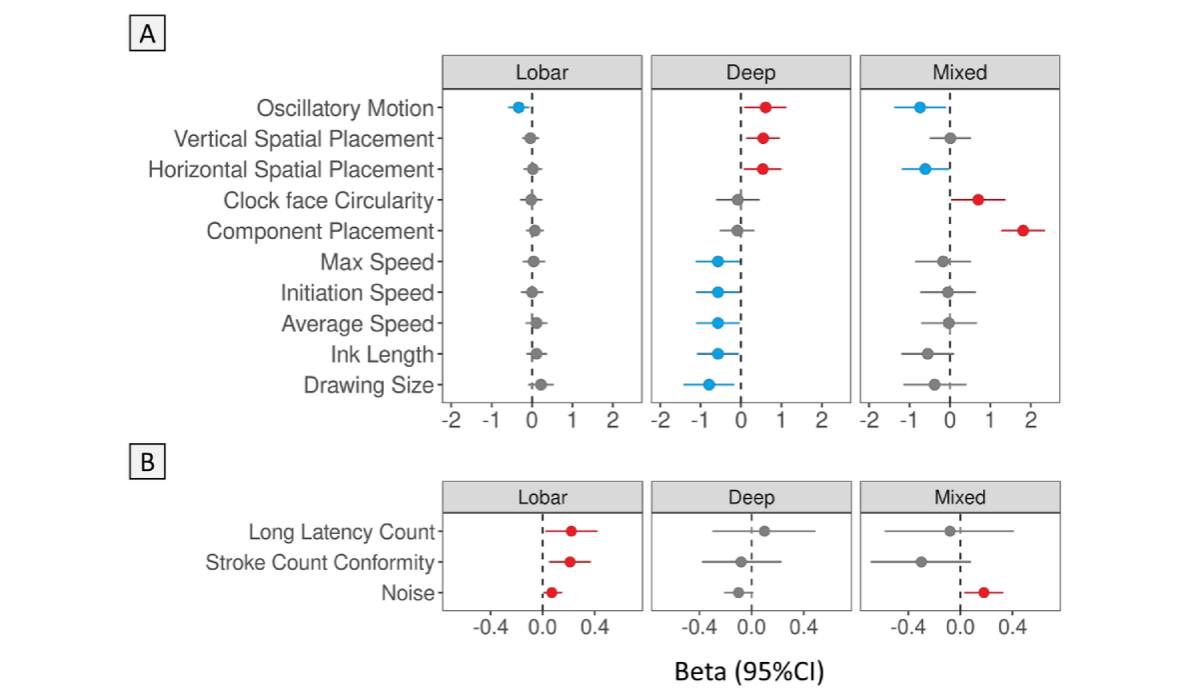
The association between facets of the DCT and the CMB subtypes. Robust multivariate linear regression models were applied with DCT scores (facets for the command and copy condition) and CMB subtypes (lobar CMB, deep CMB, and mixed [lobar+deep] CMB). The reference group were participants without CMB (n=956). The overall DCT score (percentage) was rescaled to a z score (mean 0, SD 1) after logit transformation and other DCT scores were rescaled to z scores (mean 0, SD 1) after log10 transformation. All models were adjusted for age, sex, education, WMHs, MCI, and the time difference between brain MRI and the DCT. Only facets with statistical significance for any type of CMB are shown (as indicated by the red color, with *P*<.05). The results are shown as standardized β coefficients (β) with 95% CI. CMB: cerebral microbleed; DCT: digital clock-drawing test; MCI: mild cognitive impairment; WMH: white matter hyperintensity. A: Command condition; B: Copy condition.

Next, we used all facets to explore the diagnostic potential of the DCT for CMB, with the subtypes of CMB as the predicted outcomes (lobar, deep, or mixed). In addition to including all facets in the analysis, we investigated which specific facet score had a relationship with the 3 CMB subtypes (ie, lobar-only, deep-only, and mixed; Tables S2 and S3 in Multimedia Appendix 1). Some features from the command condition were mainly associated with deep CMB, whereas the features from the copy condition were more likely to be associated with lobar CMB (Figure 1). For example, in the command condition, the features including vertical spatial placement, horizontal spatial placement, clock face circularity, component placement, max speed, initiation speed, average speed, ink length, and drawing size were associated with deep CMB but not with lobar CMB. Whereas oscillatory motion was lower among participants with lobar CMB, this feature was higher among participants with deep CMB in the command condition. In the copy condition, the features long latency count, stroke count conformity, and noise were positively associated with lobar-only CMB, but not with deep-only CMB. The mixed (lobar+deep) CMB was significantly associated with lower oscillatory motion, lower horizontal spatial placement, higher clock face circularity, and higher component placement scores in the command condition and higher noise score in the copy condition (Figure 1 and Tables S3 and S4 in Multimedia Appendix 1).

**Figure 2:**
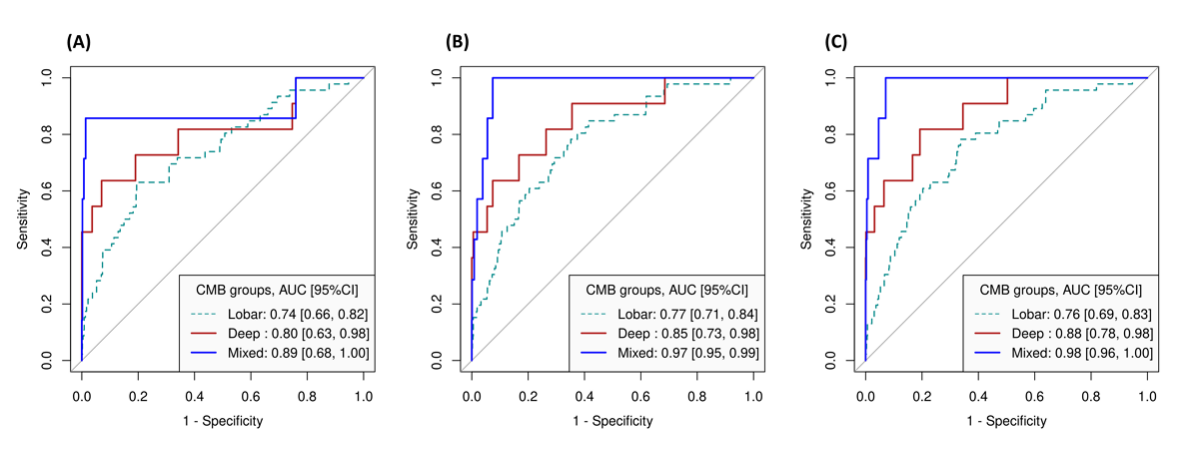
Multiclass classification of CMB evaluation with ROC curves using (A) all DCT facet scores, (B) all DCT facet scores+sex+age, and (C) all DCT facet scores+sex+age+time+MCI, where time is the time (years) difference between the brain MRI date and DCT date. AUC: area under curve; CMB: cerebral microbleed; DCT: digital clock drawing test; MCI: mild cognitive impairment; MRI: magnetic resonance imaging; ROC: receiver operating characteristic.

In the ROC curve analysis, the results indicated that DCT facet scores alone demonstrated discrimination, as evidenced by the AUCs between individuals without CMB and those with lobar CMB (AUC 0.74, 95% CI 0.66-0.82), deep CMB (AUC 0.80, 95% CI 0.63-0.98), and mixed CMB (AUC 0.89, 95% CI 0.68-1.00; Figure 2A). After adjusting for sex and age, the AUC values were further improved. The AUC (95% CI) values for individuals without CMB and those with lobar CMB, deep CMB, or mixed CMB were 0.77 (0.71-0.84), 0.85 (0.73-0.98), and 0.97 (0.95-0.99), respectively (Figure 2B). Further adjustment for the time difference between brain MRI and DCT measurement and the prevalence of MCI at the brain MRI scan yielded similar AUC values for individuals without CMB and those with lobar CMB (0.76, 95% CI 0.69-0.83), deep CMB (0.88, 95% CI 0.78-0.98), or mixed CMB (0.98, 95% CI 0.96-1.00; Figure 2C). Notably, the highest AUC was observed for the mixed CMB group, followed by a modest increase for the deep CMB group, and the lobar CMB group when compared to the no CMB group, highlighting the enhanced discriminatory ability of DCT facets in identifying different CMB subtypes after adjusting for relevant covariates.

**Table 1:** Characteristics of the study sample stratified by brain MRI^a^ CMB^b^ status.

Characteristics				Overall (N=1020)		CMB status (no=0 and yes=1)					
						No (n=956)		Yes (n=64)		*P* values^c^	
Age (years), mean (SD)				72 (7.9)		71 (7.8)		76 (8.2)		*<.001* ^d^	
Female, n (%)				585 (57)		551 (58)		34 (53)		.78	
**Education, n (%)**											.26
	High school or less			246 (24)		224 (23)		22 (34)			
	Some college			316 (31)		295 (31)		21 (33)			
	College or higher			458 (45)		437 (46)		21 (33)			
Mild cognitive impairment, n (%)				43 (4)		36 (4)		7 (11)		*.02*	
White matter hyperintensities, mean (SD)				0.0 (1.0)		–0.04 (1.0)		0.49 (1.0)		*<.001*	
**Traditional clock-drawing test^e^**											
	Command clock, median (IQR)			0 (0-3)		0 (0-2)		0 (0-3)		.36	
	Copy clock, median (IQR)			0 (0-2)		0 (0-1)		0 (0-2)		.30	
**DCT^f^, mean (SD)**											
	Overall DCT score			76 (20)		76 (20)		68 (23)		*.009*	
**Composite score, mean (SD)**											
	**Domains of command clock**										
		Drawing efficiency	62 (10)		62 (10)		60 (12)		.41		
		Simple motor	63 (8.1)		63 (8.0)		63 (9.0)		.89		
		Information processing	60 (9.9)		60 (9.9)		59 (8.8)		.83		
		Spatial reasoning	65 (17)		65 (17)		59 (20)		*.03*		
	**Domains of copy clock**										
		Drawing efficiency	61 (8.4)		61 (8.3)		59 (9.2)		*.03*		
		Simple motor	61 (6.7)		61 (6.7)		60 (7.4)		.80		
		Information processing	61 (11)		61 (11)		57 (11)		*.008*		
		Spatial reasoning	67 (18)		68 (17)		62 (21)		.06		

^a^MRI: magnetic resonance imaging.

^b^CMB: cerebral microbleed.

^c^*t* tests (*df*=1018) were applied for continuous variable comparisons between 2 groups (cerebral microbleed versus no cerebral microbleed). *χ*^2^ (female *df*=1, education *df*=2) tests were used for categorical variables, while Fisher exact tests were used in cases of low frequency. *P* values for statistical significance are shown.

^d^Significant *P* values are italicized.

^e^Due to the skewed distributions, we performed a Kruskal-Wallis rank sum test for the traditional clock-drawing test.

^f^DCT: digital clock-drawing test.

**Table 2:** The association between cerebral microbleed subtypes and the domains of the DCT^a^.

DCT		Lobar-only (n=46)		Deep-only (n=11)			Mixed (lobar+deep; n=7)	
		β (95% CI)	*P* value	β (95% CI)	*P* value	β (95% CI)		*P* value
Overall DCT score		0.05 (–0.19 to 0.29)	.67	–0.36 (–0.84 to 0.12)	.15	–0.29 (–0.90 to 0.32)		.35
**Command domains**								
	Drawing efficiency	0.20 (–0.05 to 0.45)	.12	–0.65 (–1.15 to –0.15)	*.01* ^b^	–0.28 (–0.90 to 0.35)		.39
	Simple motor	0.25 (–0.03 to 0.53)	.08	–0.86 (–1.43 to –0.30)	*.003*	0.52 (–0.19 to 1.23)		.15
	Information processing	0.04 (–0.22 to 0.31)	.75	–0.13 (–0.66 to 0.39)	.62	–0.29 (–0.95 to 0.37)		.39
	Spatial reasoning	–0.06 (–0.30 to 0.18)	.60	–0.12 (–0.59 to 0.35)	.60	–1.70 (–2.29 to –1.11)		*<.001*
**Copy domains**								
	Drawing efficiency	–0.18 (–0.45 to 0.09)	.19	–0.05 (–0.57 to 0.47)	.86	0.05 (–0.60 to 0.71)		.88
	Simple motor	0.08 (–0.20 to 0.36)	.57	–0.18 (–0.73 to 0.38)	.54	0.48 (–0.22 to 1.18)		.18
	Information processing	–0.22 (–0.49 to 0.05)	.11	–0.14 (–0.67 to 0.40)	.62	–0.09 (–0.76 to 0.58)		.79
	Spatial reasoning	–0.01 (–0.29 to 0.26)	.92	–0.22 (–0.77 to 0.32)	.43	–0.16 (–0.85 to 0.52)		.64

^a^DCT: digital clock-drawing test.

^b^Significant *P* values are italicized.

## Discussion

### Overview

This cross-sectional study in the FHS reveals the potential of the DCT as a cost-effective and objective screening tool for detecting CMB in different brain regions. Unlike the traditional CDT, the DCT offers detailed insights into cognitive function and demonstrates significant associations with CMB, particularly deep and mixed subtypes. The study highlights the limitations of traditional cognitive tests in detecting subtle brain abnormalities such as CMB and underscores the DCT’s value in early prediction of dementia risk.

### Principal Findings

To our knowledge, our study is the first to evidence that the DCT reveals detailed, nuanced, and hidden information and can serve as a potentially useful screening tool to detect the presence of CMB in different brain regions. As CMB is a risk factor for AD [28] but is accompanied by no or very subtle clinical symptoms, it is costly for clinicians to use neuroimaging to detect CMB. In comparison, the DCT is more cost-effective and can be completed by patients within a couple of minutes with minimal assistance. Another advantage of the DCT is that it objectively captures detailed brain functions through automated digital collection and analysis, while the manual traditional counterpart depends on the subjective assessment by trained testers. Using the DCT may be useful for in future clinical practice for early screening and detection of CMB, triggering interventions that can delay the progress of the disease or prevent AD onset [35].

### Comparison to Prior Work

Whereas the traditional CDT has low sensitivity and specificity to screen or diagnose participants with CMB, the DCT scores were significantly associated with CMB in our study, especially deep and mixed CMB, including across 3 different measurement levels (ie, overall score, domains, and facets of the DCT). Previous studies have reported inconsistent associations between CMB and the traditional CDT. For example, whereas 1 study found that CMB were a risk factor for low performance on the CDT [36], 2 others did not find a relationship [37,38]. Another study that did not exclude participants with dementia illustrated that lobar, but not deep, CMB were associated with CDT [39]. It is possible that CDT’s crude measurements contain mixtures of different cognitive functions that cannot be broken down into more detailed measurements such as facet scores of DCT and thus may not be able to clearly detect fine brain abnormalities like CMB.

### Strengths and Limitations

There were several strengths in our study. First, in our study, the diagnostic discriminability using the DCT facet scores was able to differentiate those with and without CMB, especially the mixed subtype, independent from MCI. Second, the DCT is simple, can be self-administered, and could serve as a screening test before administering costly neuroimaging tests. Third, our study found that participants with lobar CMB and deep CMB had different DCT performance patterns. Whereas the command condition was more strongly associated with deep or mixed CMB, the copy condition may be more associated with lobar CMB. Since deep but not lobar CMB have been identified as risk factors for dementia development in previous studies [28], the DCT may be a valuable tool for the early prediction of dementia risk. By capturing multiple facets of cognitive function as well as their fine-grained interrelations, the DCT affords substantially more sensitive analyses compared to typical measures of domain-specific cognitive functions that are observed in isolation (or aggregated in sum scores that overlook fine-grained interrelations).

This study had several limitations. First, the sample size of deep and mixed CMB cases is relatively small, potentially limiting the generalizability of findings and statistical power to detect associations. Future studies should aim for larger and more diverse cohorts. Second, the associative study design restricts the ability to establish causal relationships between variables, despite efforts to control for confounding factors. Future research could benefit from longitudinal or interventional approaches to explore causality further. Lastly, the lack of diversity in the FHS cohort may limit the applicability of findings to broader populations. Future studies should strive to include more diverse cohorts to enhance generalizability and reduce potential biases.

### Future Directions

Large cohorts with multiethnicities should be used to confirm that the DCT and other digital tools detect CMB and similar pathologies in different brain regions and can serve as a cost-effective screening tool to better identify people at risk earlier in the preclinical stages of the disease [11]. More importantly, user-oriented assessment devices such as the DCT may promote objectivity and equity within public health research, especially in underserved populations.

## Appendix

Multimedia Appendix 1 The descriptions of each subdomain of digital clock-drawing test (DCT) scores.

## Abbreviations

AD: Alzheimer disease
AUC: area under the curve
CDT: clock-drawing test
CMB: cerebral microbleed
DCT: digital clock-drawing test
FHS: Framingham Heart Study
MCI: mild cognitive impairment
MRI: magnetic resonance imaging
ROC: receiver operating characteristic
WMH: white matter hyperintensity

## Data Availability

The data sets generated or analyzed during this study are available through the Framingham Heart Study central database. For detailed information on data access, please visit the Framingham Heart Study Brain Aging Program (FHS-BAP) Data Access Portal [40].
