# Supplementary material for: Cerebral Microbleeds in Different Brain Regions and Their Associations With the Digital Clock-Drawing Test: Secondary Analysis of the Framingham Heart Study
Source: J Med Internet Res. 2024 Jul 29;26:e45780. doi: 10.2196/45780 (PMC11319892; doi:10.2196/45780)
Supplement: Multimedia Appendix 1 [file jmir_v26i1e45780_app1.docx]

**Table S1.** The descriptions of each subdomain of digital clock drawing test (DCT) scores.

| Domain-specific scores* | Metric Type | Description |
| --- | --- | --- |
| Drawing Efficiency | **Domain** | This considers metrics such as pen stroke speeds and oscillatory motion and can be helpful in parsing out motor from non-motor cognitive functions. |
| Drawing Process Efficiency | Facet | A relative measure that combines Ink Length and Total Time on the clock drawing. |
| Drawing Size | Facet | The size, in millimeters, of the clock face circle of the clock. |
| Ink Length | Facet | The sum, in millimeters, of all pen stroke lengths used in the clock drawing. |
| Noise | Facet | A measure of the drawing that includes non-standard pen strokes, cross-outs, and overwriting on the clock drawing. |
| Stroke Count Conformity | Facet | The deviation from the expected number of pen strokes in the clock drawing. |
| Total Time | Facet | The total time, in seconds, spent completing the clock drawing measured from the first touch of the pen on the paper to the last pen lift off the paper. |
| Information Processing | **Domain** | This considers metrics such as absolute and relative duration of latencies, number of pauses, and relative time spent thinking versus actively drawing with pen on the paper. |
| Average Latency | Facet | The average duration of the latencies between each pen stroke of the clock drawing. |
| Latency Variability | Facet | The variability in the latencies throughout the drawing process of the clock. |
| Longest Latency | Facet | The duration of the longest latency in the clock drawing. |
| Long Latency Count | Facet | The total number of latencies in clock drawing that are notably longer than the normative sample standard. |
| Relative Long Latency | Facet | A measure of the differences among the average latency and the longer latencies within the drawing of the command clock. |
| Percent Think Time | Facet | The percentage of the test time spent "thinking" (i.e., holding the pen off the page but not actively drawing), measured from the first touch of the pen on the paper to the last pen lift off the paper, on the clock drawing. |
| Simple Motor | **Domain** | This considers metrics such as absolute and relative duration of latencies, number of pauses, and relative time spent thinking versus actively drawing with pen on the paper. |
| Average Speed | Facet | The average speed of the pen for all pen strokes used during the drawing of the clock face. |
| Initiation Speed | Facet | The speed of the pen when beginning to draw the clock face. |
| Max Speed | Facet | The maximum speed of the pen on the page during the drawing of the clock face of the clock drawing. |
| Termination Speed | Facet | The speed of the pen when finishing the clock face of the clock drawing. |
| Oscillatory Motion | Facet | A measure of how much the motion of the pen deviates from a smooth pen motion during the drawing process of the clock drawing. |
| Percent Ink Time | Facet | The percentage of the test time spent actively drawing with the pen on the paper for the clock drawing. |
| Spatial Reasoning | **Domain** | This considers metrics pertaining to the geometric properties of the drawing including the circularity of the clock circle, placement of clock components, and drawing placement on the page. |
| Clock face Circularity | Facet | A measure of the roundness of the clock face circle on the clock drawing. |
| Component Placement | Facet | A measure of the spatial relationships among the drawing components on the clock. |
| Horizontal Spatial Placement | Facet | A measure of the horizontal position of the drawing on the page |
| Vertical Spatial Placement | Facet | A measure of the vertical position of the drawing on the page. |

^*^Domain-specific scores are produced for both command and copy versions of the task in addition to facets for each domain-specific score.

**Table S2.** Sensitivity analysis of the association between CMB subtypes and the domains of the digital clock-drawing test (DCT).

| **DCT** | **All CMB**  (n = 71) | |  | **Lobar only**  (n = 48) | |  | **Deep only**  (n = 14) | |  | **Mixed**  (n = 9) | |
| --- | --- | --- | --- | --- | --- | --- | --- | --- | --- | --- | --- |
|  | β [95%CI] | P value |  | β [95%CI] | P value |  | β [95%CI] | P value |  | β [95%CI] | P value |
| **Overall DCT score** | -0.04 [-0.24, 0.15] | .67 |  | 0.07 [-0.16, 0.30] | .54 |  | -0.36 [-0.77, 0.06] | 0.093 |  | -0.13 [-0.65, 0.40] | 0.64 |
| **Command Domains** |  |  |  |  |  |  |  |  |  |  |  |
| Drawing Efficiency | 0.05 [-0.15, 0.25] | .62 |  | 0.20 [-0.03, 0.44] | .087 |  | -0.60 [-1.03, -0.17] | 0.006 |  | 0.02 [-0.51, 0.55] | 0.95 |
| Simple Motor | 0.06 [-0.17, 0.30] | .58 |  | 0.24 [-0.03, 0.52] | .079 |  | -0.83 [-1.32, -0.33] | 0.001 |  | 0.51 [-0.11, 1.12] | 0.11 |
| Information Processing | -0.01 [-0.22, 0.20] | .91 |  | 0.03 [-0.22, 0.28] | .80 |  | -0.02 [-0.48, 0.43] | 0.92 |  | -0.33 [-0.89, 0.23] | 0.25 |
| Spatial Reasoning | -0.19 [-0.38, 0.01] | .060 |  | -0.03 [-0.26, 0.20] | .80 |  | -0.38 [-0.78, 0.03] | 0.070 |  | -1.25 [-1.76, -0.74] | <0.001 |
| **Copy Domains** |  |  |  |  |  |  |  |  |  |  |  |
| Drawing Efficiency | -0.06 [-0.27, 0.15] | .58 |  | -0.12 [-0.37, 0.13] | .35 |  | 0.01 [-0.44, 0.46] | 0.95 |  | 0.19 [-0.37, 0.75] | 0.51 |
| Simple Motor | 0.05 [-0.18, 0.28] | .66 |  | 0.10 [-0.18, 0.37] | .49 |  | -0.27 [-0.76, 0.23] | 0.29 |  | 0.36 [-0.25, 0.97] | 0.25 |
| Information Processing | -0.19 [-0.40, 0.02] | .083 |  | -0.20 [-0.46, 0.05] | .11 |  | -0.15 [-0.61, 0.31] | 0.51 |  | -0.12 [-0.69, 0.45] | 0.68 |
| Spatial Reasoning | -0.04 [-0.27, 0.18] | .71 |  | 0.00 [-0.26, 0.27] | .97 |  | -0.18 [-0.65, 0.30] | 0.47 |  | -0.04 [-0.64, 0.55] | 0.89 |

Robust multivariate linear regression models were applied with digital clock-drawing test (DCT) scores (overall score and domains) and CMB subtypes (all CMB, lobar only, deep only, any deep). The reference group were participants without CMBs (n = 956). Participants with stroke and dementia were included in the sensitivity analysis. The overall DCT score (percentage) was rescaled to a z-score (mean = 0, SD = 1) after logit transformation and other DCT scores were rescaled to z-scores (mean = 0, SD = 1) after logit transformation. All models were adjusted for age, sex, education, white matter hyperintensities (WMHs), history of dementia, history of stroke, and time difference between brain MRI and the DCT. Results are shown as standardized Beta-coefficients (β) with 95% confidence intervals (CI). *P-*values for statistical significance are indicated.

**Table S3.** The association between CMB and the facets of the DCT under the command condition.

| **DCT facets  (command condition)** | **All CMB** (n = 64) | |  | **Lobar only** (n = 46) | |  | **Deep only** (n = 11) | |  | **Mixed** (n = 7) | |
| --- | --- | --- | --- | --- | --- | --- | --- | --- | --- | --- | --- |
|  | β [95%CI] | P value |  | β [95%CI] | P value |  | β [95%CI] | P value |  | β [95%CI] | P value |
| Drawing Efficiency |  |  |  |  |  |  |  |  |  |  |  |
| Drawing Process Efficiency | -0.01 [-0.25, 0.24] | .95 |  | 0.18 [-0.10, 0.46] | .22 |  | -0.50 [-1.07, 0.06] | .08 |  | -0.40 [-1.11, 0.31] | .27 |
| Drawing Size | 0.02 [-0.25, 0.30] | .86 |  | 0.22 [-0.09, 0.53] | .17 |  | -0.79 [-1.42, -0.17] | **.012** |  | -0.38 [-1.15, 0.40] | .35 |
| Ink Length | -0.05 [-0.27, 0.18] | .68 |  | 0.11 [-0.14, 0.37] | .39 |  | -0.57 [-1.09, -0.06] | **.030** |  | -0.55 [-1.20, 0.10] | .10 |
| Noise | -0.01 [-0.12, 0.09] | .80 |  | 0.02 [-0.10, 0.14] | .76 |  | -0.14 [-0.38, 0.10] | .25 |  | -0.04 [-0.34, 0.25] | .78 |
| Stroke Count Conformity | -0.03 [-0.11, 0.05] | .51 |  | -0.02 [-0.12, 0.07] | .61 |  | -0.12 [-0.31, 0.07] | .23 |  | 0.12 [-0.12, 0.36] | .34 |
| Total Time | 0.01 [-0.15, 0.17] | .88 |  | -0.02 [-0.20, 0.17] | .86 |  | 0.13 [-0.24, 0.49] | .49 |  | -0.05 [-0.51, 0.42] | .85 |
| Simple Motor |  |  |  |  |  |  |  |  |  |  |  |
| Average Speed | -0.04 [-0.27, 0.20] | .75 |  | 0.11 [-0.16, 0.38] | .43 |  | -0.57 [-1.11, -0.03] | **.039** |  | -0.03 [-0.71, 0.66] | .94 |
| Initiation Speed | -0.11 [-0.34, 0.13] | .38 |  | 0.00 [-0.27, 0.27] | .99 |  | -0.57 [-1.11, -0.03] | **.038** |  | -0.05 [-0.73, 0.64] | .90 |
| Max Speed | -0.10 [-0.34, 0.14] | .42 |  | 0.04 [-0.23, 0.32] | .76 |  | -0.57 [-1.12, -0.03] | **.041** |  | -0.17 [-0.86, 0.52] | .63 |
| Termination Speed | 0.02 [-0.20, 0.24] | .88 |  | 0.11 [-0.15, 0.36] | .42 |  | -0.36 [-0.87, 0.15] | .17 |  | 0.11 [-0.54, 0.75] | .75 |
| Oscillatory Motion | -0.24 [-0.46, -0.02] | **.035** |  | -0.33 [-0.59, -0.07] | **.012** |  | 0.61 [ 0.09, 1.12] | **.021** |  | -0.74 [-1.38, -0.10] | **.025** |
| Percent Ink Time | 0.12 [-0.14, 0.39] | .37 |  | 0.24 [-0.07, 0.55] | .13 |  | -0.06 [-0.66, 0.55] | .85 |  | -0.29 [-1.05, 0.47] | .45 |
| Information Processing |  |  |  |  |  |  |  |  |  |  |  |
| Average Latency | 0.06 [-0.10, 0.22] | .49 |  | -0.03 [-0.21, 0.15] | .75 |  | 0.30 [-0.07, 0.66] | .11 |  | 0.27 [-0.19, 0.72] | .25 |
| Latency Variability | 0.00 [-0.12, 0.12] | .99 |  | -0.02 [-0.16, 0.13] | .84 |  | 0.02 [-0.27, 0.31] | .88 |  | 0.11 [-0.25, 0.47] | .57 |
| Longest Latency | -0.02 [-0.14, 0.10] | .76 |  | -0.03 [-0.17, 0.11] | .69 |  | 0.07 [-0.21, 0.35] | .65 |  | -0.05 [-0.40, 0.30] | .79 |
| Long Latency Count | -0.01 [-0.21, 0.19] | .91 |  | -0.02 [-0.25, 0.22] | .88 |  | -0.23 [-0.70, 0.24] | .34 |  | 0.39 [-0.20, 0.98] | .19 |
| Relative Long Latency | -0.01 [-0.14, 0.11] | .84 |  | -0.03 [-0.17, 0.12] | .70 |  | -0.02 [-0.30, 0.26] | .90 |  | 0.14 [-0.21, 0.50] | .43 |
| Percent Think Time | -0.12 [-0.39, 0.14] | .37 |  | -0.24 [-0.55, 0.07] | .13 |  | 0.06 [-0.55, 0.66] | .85 |  | 0.29 [-0.47, 1.05] | .45 |
| Spatial Reasoning |  |  |  |  |  |  |  |  |  |  |  |
| Clock face Circularity | 0.04 [-0.19, 0.28] | .71 |  | -0.02 [-0.29, 0.25] | .88 |  | -0.08 [-0.61, 0.46] | .78 |  | 0.70 [ 0.03, 1.37] | **.040** |
| Component Placement | 0.12 [-0.07, 0.31] | .20 |  | 0.07 [-0.15, 0.29] | .60 |  | -0.09 [-0.52, 0.33] | .70 |  | 1.81 [ 1.27, 2.35] | **< .001** |
| Horizontal Spatial Placement | 0.01 [-0.19, 0.22] | .89 |  | 0.02 [-0.21, 0.25] | .87 |  | 0.54 [ 0.07, 1.00] | **.025** |  | -0.61 [-1.19, -0.03] | **.041** |
| Vertical Spatial Placement | 0.04 [-0.14, 0.21] | .69 |  | -0.04 [-0.24, 0.17] | .72 |  | 0.55 [ 0.13, 0.96] | **.010** |  | 0.01 [-0.50, 0.52] | .97 |

Robust multivariate linear regression models were applied with digital clock-drawing test (DCT) scores (facets for the command condition) and CMB subtypes (all CMB, lobar only, deep only, any deep). The reference group was no CMB (n = 956). DCT scores were rescaled to z-scores (mean = 0, SD = 1). All models were adjusted for age, sex, education, MCI, white matter hyperintensities (WMHs), and time difference between brain MRI and the DCT. Results are shown as standardized Beta-coefficients (B) with 95% confidence intervals (CI).

**Table S4.** The association between CMB and the facets of the DCT under the copy condition.

| **DCT facets**  **(copy condition)** | **All CMB** (n = 64) | |  | **Lobar only** (n = 46) | |  | **Deep only** (n = 11) | |  | **Any Deep** (n = 18) | |
| --- | --- | --- | --- | --- | --- | --- | --- | --- | --- | --- | --- |
|  | β [95%CI] | P value |  | β [95%CI] | P value |  | β [95%CI] | P value |  | β [95%CI] | P value |
| Drawing Efficiency |  |  |  |  |  |  |  |  |  |  |  |
| Drawing Process Efficiency | -0.17 [-0.42, 0.07] | .17 |  | -0.13 [-0.42, 0.15] | .37 |  | -0.34 [-0.91, 0.23] | .24 |  | -0.10 [-0.81, 0.62] | .79 |
| Drawing Size | 0.01 [-0.24, 0.26] | .95 |  | 0.06 [-0.23, 0.35] | .70 |  | -0.02 [-0.60, 0.55] | .93 |  | -0.23 [-0.95, 0.50] | .54 |
| Ink Length | -0.02 [-0.22, 0.18] | .84 |  | 0.05 [-0.18, 0.28] | .65 |  | -0.23 [-0.69, 0.22] | .32 |  | -0.16 [-0.73, 0.41] | .58 |
| Noise | 0.08 [-0.05, 0.21] | .25 |  | 0.21 [ 0.05, 0.37] | **.008** |  | -0.08 [-0.38, 0.23] | .63 |  | -0.30 [-0.69, 0.08] | .12 |
| Stroke Count Conformity | 0.04 [-0.02, 0.10] | .19 |  | 0.07 [ 0.00, 0.15] | .045 |  | -0.10 [-0.21, 0.01] | .086 |  | 0.18 [ 0.03, 0.33] | **.021** |
| Total Time | 0.16 [-0.01, 0.32] | .061 |  | 0.18 [-0.01, 0.37] | .069 |  | 0.16 [-0.21, 0.54] | .40 |  | -0.02 [-0.49, 0.45] | .95 |
| Simple Motor |  |  |  |  |  |  |  |  |  |  |  |
| Average Speed | -0.11 [-0.32, 0.11] | .34 |  | -0.07 [-0.33, 0.18] | .57 |  | -0.29 [-0.78, 0.21] | .26 |  | 0.09 [-0.54, 0.71] | .78 |
| Initiation Speed | -0.12 [-0.34, 0.10] | .27 |  | -0.09 [-0.34, 0.17] | .50 |  | -0.26 [-0.77, 0.24] | .31 |  | -0.06 [-0.70, 0.58] | .85 |
| Max Speed | -0.21 [-0.44, 0.02] | .08 |  | -0.19 [-0.46, 0.09] | .18 |  | -0.41 [-0.94, 0.13] | .14 |  | 0.06 [-0.61, 0.73] | .86 |
| Termination Speed | -0.07 [-0.27, 0.12] | .47 |  | -0.05 [-0.29, 0.18] | .65 |  | -0.25 [-0.70, 0.21] | .29 |  | 0.18 [-0.39, 0.75] | .54 |
| Oscillatory Motion | -0.24 [-0.46, -0.01] | **.037** |  | -0.21 [-0.47, 0.04] | .10 |  | -0.09 [-0.60, 0.41] | .72 |  | -0.56 [-1.19, 0.08] | .09 |
| Percent Ink Time | -0.02 [-0.28, 0.25] | .91 |  | -0.04 [-0.34, 0.27] | .82 |  | 0.11 [-0.51, 0.72] | .73 |  | -0.17 [-0.95, 0.60] | .66 |
| Information Processing |  |  |  |  |  |  |  |  |  |  |  |
| Average Latency | 0.16 [-0.04, 0.36] | .11 |  | 0.15 [-0.07, 0.38] | .19 |  | 0.20 [-0.26, 0.65] | .40 |  | 0.08 [-0.49, 0.65] | .78 |
| Latency Variability | 0.10 [-0.05, 0.25] | .20 |  | 0.11 [-0.06, 0.29] | .20 |  | 0.08 [-0.26, 0.42] | .65 |  | 0.00 [-0.43, 0.42] | .98 |
| Longest Latency | 0.09 [-0.05, 0.23] | .20 |  | 0.09 [-0.07, 0.26] | .27 |  | 0.14 [-0.19, 0.46] | .41 |  | -0.01 [-0.42, 0.40] | .96 |
| Long Latency Count | 0.16 [-0.01, 0.34] | .061 |  | 0.22 [ 0.02, 0.42] | **.033** |  | 0.10 [-0.30, 0.49] | .63 |  | -0.08 [-0.58, 0.41] | .74 |
| Relative Long Latency | 0.10 [-0.06, 0.26] | .23 |  | 0.14 [-0.05, 0.32] | .14 |  | -0.04 [-0.40, 0.32] | .83 |  | 0.05 [-0.40, 0.51] | .82 |
| Percent Think Time | 0.02 [-0.25, 0.28] | .91 |  | 0.04 [-0.27, 0.34] | .82 |  | -0.11 [-0.72, 0.51] | .73 |  | 0.17 [-0.60, 0.95] | .66 |
| Spatial Reasoning |  |  |  |  |  |  |  |  |  |  |  |
| Clock face Circularity | -0.02 [-0.29, 0.24] | .86 |  | -0.04 [-0.34, 0.27] | .82 |  | -0.08 [-0.68, 0.52] | .80 |  | 0.19 [-0.56, 0.95] | .62 |
| Component Placement | 0.10 [-0.12, 0.32] | .37 |  | 0.15 [-0.11, 0.40] | .27 |  | -0.18 [-0.69, 0.32] | .48 |  | 0.36 [-0.28, 0.99] | .27 |
| Horizontal Spatial Placement | 0.12 [-0.12, 0.35] | .34 |  | 0.03 [-0.25, 0.30] | .84 |  | 0.47 [-0.08, 1.03] | .092 |  | 0.27 [-0.41, 0.96] | .44 |
| Vertical Spatial Placement | 0.07 [-0.19, 0.33] | .59 |  | -0.01 [-0.30, 0.29] | .96 |  | 0.46 [-0.13, 1.06] | .13 |  | 0.22 [-0.51, 0.96] | .56 |

Robust multivariate linear regression models were applied with digital clock-drawing test (DCT) scores (facets for the copy condition) and CMB subtypes (all CMB, lobar only, deep only, any deep). The reference group was no CMB (n = 956). DCT scores were rescaled to z-scores (mean = 0, SD = 1). All models were adjusted for age, sex, education, MCI, white matter hyperintensities (WMHs), and time difference between brain MRI and the DCT. Results are shown as standardized Beta-coefficients (B) with 95% confidence intervals (CI).

**Table S5**. The CMB numbers distribution among three CMB groups (Lobar, Deep and Mix).

| Groups | CMB numbers | | | | | | | |
| --- | --- | --- | --- | --- | --- | --- | --- | --- |
|  | **0** | **1** | **2** | **3** | **6** | **7** | **13** | **26** |
| no CMB | 956 | 0 | 0 | 0 | 0 | 0 | 0 | 0 |
| Lobar | 0 | 40 | 3 | 2 | 0 | 0 | 0 | **1** |
| Deep | 0 | 10 | 1 | 0 | 0 | 0 | 0 | 0 |
| Mix | 0 | 0 | 1 | 3 | **1** | **1** | **1** | 0 |
